# Supplementary material for: Characterization of the Subclinical Infection of Porcine Deltacoronavirus in Grower Pigs under Experimental Conditions
Source: Viruses. 2022 Sep 28;14(10):2144. doi: 10.3390/v14102144 (PMC9611937; doi:10.3390/v14102144)
Supplement: Supplementary file 1 [file viruses-14-02144-s001.zip › viruses-1930172-supplementary.pdf]

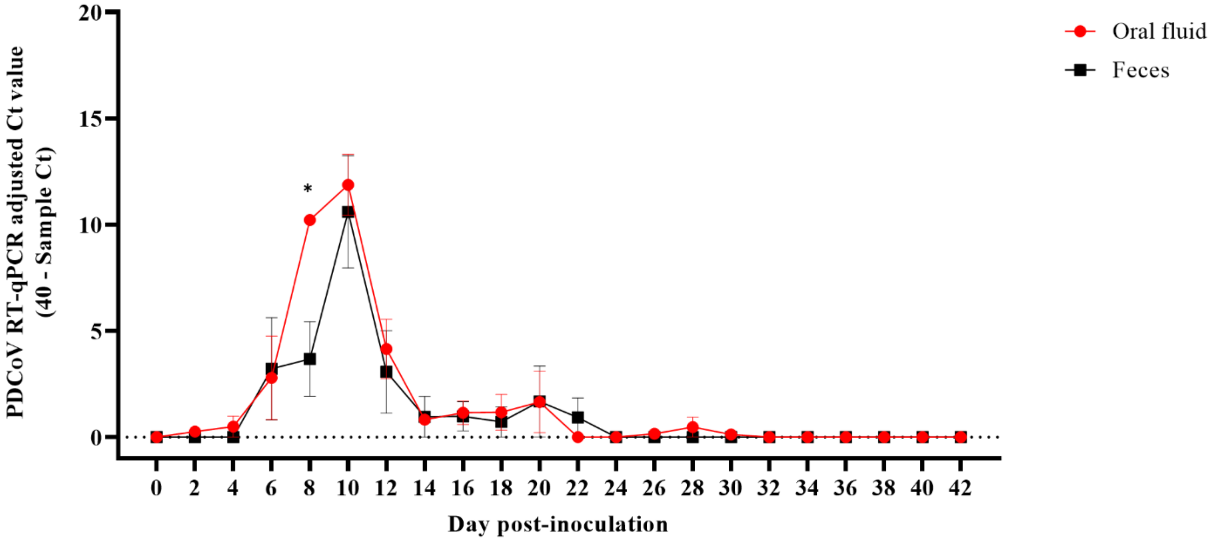

Figure S1. Comparison porcine deltacoronavirus (PDCoV RNA) shedding levels (Ct values) via RT-qPCR in pen-based feces and oral fluid collected between day post-inoculation (DPI) 0 to 42 from PDCoV-inoculated 7-week-old grower pigs. This graph represents the average data of 40-sample Ct values on the Y-axis and day post-inoculation in the X-axis. Red line denotes detection in pen-based oral fluids, while black line denotes detection in feces. Error bars represent the standard error of the mean (SEM), and * denotes statistical significance ($p < 0.05$).
